# Association of TNF-alpha, IL-6 and IL-1beta gene polymorphisms with polycystic ovary syndrome: a meta-analysis

**DOI:** 10.1186/s12863-015-0165-4

**Published:** 2015-01-30

**Authors:** Renyong Guo, Ying Zheng, Jiezuan Yang, Nengneng Zheng

**Affiliations:** Department of Laboratory Medicine, First Affiliated Hospital, College of Medicine, Zhejiang University, Hangzhou, Zhejiang 310003 China; Department of Gynecology and Obstetrics, Tongde Hospital of Zhejiang Province, Hangzhou, Zhejiang 310012 China; State Key Laboratory for Diagnosis and Treatment of Infectious Disease, First Affiliated Hospital, College of Medicine, Zhejiang University, Hangzhou, 310003 China

**Keywords:** Polycystic ovary syndrome, *TNF-alpha*, *IL-6*, *IL-1beta*, Polymorphism, Meta-analysis

## Abstract

**Background:**

Several studies on the association of *TNF-alpha (−308 G/A)*, *IL-6 (−174 G/C)* and *IL-1beta (−511 C/T)* polymorphisms with polycystic ovary syndrome (PCOS) risk have reported conflicting results. The aim of the present study was to assess these associations by meta-analysis.

**Results:**

A total of 14 eligible articles (1665 cases/1687 controls) were included in this meta-analysis. The results suggested that there was no obvious association between the *TNF-alpha (−308 G/A)* polymorphism and PCOS in the overall population or subgroup analysis by ethnicity, Hardy–Weinberg equilibrium (HWE) in controls, genotyping method, PCOS diagnosis criteria, and study sample size. Also, no obvious association was found between the *TNF-alpha (−308 G/A)* polymorphism and obesity in patients with PCOS (body mass index [BMI] ≥ 25 kg/m^2^ vs. BMI < 25 kg/m^2^). Regarding the *IL-6 (−174 G/C)* polymorphism, also no association was found in the overall population in heterozygote comparison, dominant model, and recessive model. Even though an allelic model (odds ratio [OR] = 0.63, 95% confidence interval [CI] = 0.41–0.96) and a homozygote comparison (OR = 0.52, 95% CI = 0.30–0.93) showed that the *IL-6 (−174 G/C)* polymorphism was marginally associated with PCOS. Further subgroup analysis suggested that the effect size was not significant among HWE in controls (sample size ≤ 200) and genotyping method of pyrosequencing under all genetic models. Similarly, there was no association between the *IL-1beta (−511 C/T)* polymorphism and PCOS in the overall population or subgroup analysis under all genetic models. Furthermore, no significant association was found between the *IL-1beta (−511 C/T)* polymorphism and several clinical and biochemical parameters in patients with PCOS.

**Conclusions:**

The results of this meta-analysis suggest that the *TNF-alpha (−308 G/A)*, *IL-6 (−174 G/C)*, and *IL-1beta (−511 C/T)* polymorphisms may not be associated with PCOS risk. However, further case–control studies with larger sample sizes are needed to confirm our results.

**Electronic supplementary material:**

The online version of this article (doi:10.1186/s12863-015-0165-4) contains supplementary material, which is available to authorized users.

## Background

Polycystic ovary syndrome (PCOS) is a common and complex endocrine disorder leading to reproductive dysfunction in females, with a prevalence of 5%–10% [1]. Hyperandrogenism, oligo/anovulation, polycystic ovaries, hyperinsulinemia, and obesity are main manifestations of PCOS with a subsequent increased risk for type 2 diabetes [2]. Although the etiology of PCOS remains controversial, interactions of hereditary and environmental factors are reportedly associated with PCOS [3].

Recent studies have shown that chronic low grade inflammation is closely related to the incidence of PCOS [4]. Pro-inflammatory cytokines, such as tumor necrosis factor alpha (TNF-alpha), interleukin (IL)-6, and IL-1, are prominent mediators of inflammation and have been confirmed to be elevated in at least a subgroup of women with PCOS [5-7]. The corpus luteum secretes TNF-alpha and the levels of immunoreactive TNF-alpha vary throughout the menstrual cycle [8]. TNF-alpha, IL-6, and IL-1 are presumed to play pivotal roles in reproductive physiology, including regulation of ovarian steroid production, follicular maturation, and the processes of ovulation, fertilization, and implantation– parameters all affected in women with PCOS [9,10].

The *TNF-alpha* gene resides within the class III region of the major histocompatibility complex and is located on the short arm of chromosome 6 (6p21.3). A single nucleotide polymorphism (SNP) located at position −308 in the promoter region of the *TNF-alpha* gene gives rise to a G-A exchange, which has been associated with elevated serum TNF-alpha concentrations in certain clinical states [5,11]. The human *IL-6* gene is located at chromosome 7p21-24 and has an upstream 303 bp promoter. A SNP, which results in exchange of G-C at position −174 in the promoter region of the *IL-6* gene, has been found to influence its transcription rate [12]. The *IL-1* gene cluster on chromosome 2q12-13 contains three related genes, *IL-1alpha*, *IL-1beta*, and *IL-1RN*. A common C/T SNP of the *IL-1beta* promoter at position −511 has been found to correspond with altered IL-1beta protein expression both *in vitro* and *in vivo* [13].

Recently, a number of research groups have evaluated the usefulness of the *TNF-alpha (−308 G/A)*, *IL-6 (−174 G/C)*, and *IL-1beta (−511 C/T)* polymorphisms as potential susceptibility factors for PCOS. However, the results of published studies are inconclusive and even controversial [7,14-26], which could be due to differences in the studied populations and limited sample sizes. Therefore, in this study, a meta-analysis of 14 eligible articles was performed to clarify these inconsistences and provide more conclusive results.

## Methods

### Publication search and inclusion criteria

To identify all published reports on the association between *TNF-alpha (−308 G/A)*, *IL-6 (−174 G/C)*, and *IL-1beta (−511 C/T)* polymorphisms and PCOS risk, we performed a systematic literature search of PubMed, ISI Web of Science, Elsevier Science Direct, China Biology Medical Literature Database, China National Knowledge Infrastructure, and Wanfang online libraries using the terms “polymorphism or variant or mutation” and “polycystic ovary syndrome or PCOS” and “tumor necrosis factor alpha or TNF-alpha or interleukin-6 or IL-6 or interleukin-1beta or IL-1beta” without any language restrictions. We also conducted a manual search of references in the individual articles to identify other potential publications. All clearly irrelevant studies, case reports, editorials, and review articles were excluded. The literature search was updated on September 15, 2014. This meta-analysis was conducted and reported according to the Preferred Reporting Items for Systematic Reviews and Meta-Analysis (PRISMA) Statement [27] (see the checklist included in Additional file 1).

Eligible studies were selected according to the following explicit inclusion criteria: (1) evaluated the association between *TNF-alpha (−308 G/A)*, *IL-6 (−174 G/C)* and *IL-1beta (−511 C/T)* polymorphisms and susceptibility to PCOS; (2) case–control studies based on unrelated individuals; (3) all patients met the diagnostic criteria for PCOS; (4) must provide sufficient genotype data to calculate odds ratios (ORs) and 95% confidence intervals (CIs). Articles that did not meet our inclusion criteria were excluded. When a study reported the results on different ethnicities, we treated them independently.

### Data extraction

Two authors independently extracted all data based on the inclusion criteria listed above. Any disagreement was subsequently resolved by consensus with a third author. The following items were collected from each study: the first author’s name, year of publication, source of publication, ethnicity of the population, sample size, PCOS diagnostic criteria, genotyping method, genotype frequencies, and probability (*p*) value for the Hardy–Weinberg equilibrium (HWE) in controls. If original genotype frequency data were unavailable in relevant articles, a request for additional data was sent to the corresponding author.

### Quality assessment

Two reviewers independently assessed the quality of every included study according to the Newcastle-Ottawa Scale (NOS) (www.ohri.ca/programs/clinical_epidemiology/oxford.asp). This scale contains nine items (1 point for each) in three parts relating to selection, comparability and ascertainment of exposure. A score of five or more was defined as having high quality; otherwise, the study was regarded as "low quality" [28].

### Statistical analysis

Crude ORs with 95% CIs were used to assess the association between *TNF-alpha (−308 G/A)*, *IL-6 (−174 G/C)* and *IL-1beta (−511 C/T)* polymorphisms with PCOS risk. Analysis of polymorphisms was conducted in at least two studies. The pooled OR was calculated for the allele model, homozygote comparison, heterozygote comparison, dominant model, and recessive model. The Z test was used to estimate the statistical significance of pooled ORs and a *p*-value < 0.05 was considered statistically significant. Genotype frequencies of healthy controls were tested for HWE using the *χ*^2^ test. The Cochran Q test and I^2^ test were used to evaluate potential heterogeneity between studies. Significant heterogeneity was indicated by *p* < 0.10 for the Q test or I^2^ test of > 50% [29]. The pooled ORs were analyzed using the random effects model [30]. Otherwise, the fixed effects model [31] was selected. Subgroup analyses were conducted to explore reasons for heterogeneity. In order to evaluate the influence of single studies on the overall estimate, sensitivity analysis was performed. Potential publication bias was diagnosed statistically via the Begg’s test and Egger’s test. The presence of publication bias was indicated by a *p*-value < 0.05 [32,33]. Data management and analysis were performed using STATA statistical software (version 12.0; Stata Corporation, College Station, TX, USA).

## Results

### Characteristics of included studies

**Figure 1:**
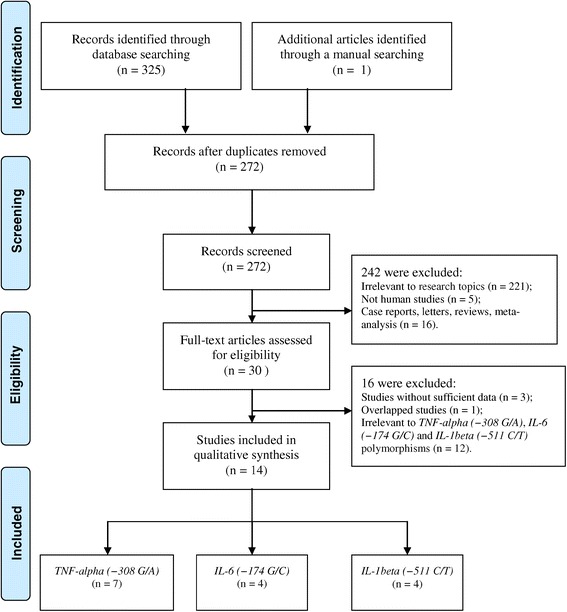
**Flow diagram of the study selection process.**

The literature search identified a total of 326 potentially relevant papers. There were 272 potentially relevant papers after duplicates were removed. After review of the titles and abstracts of all articles, 242 were excluded; full texts were also reviewed and 16 articles were further excluded. Finally, a total of 14 eligible articles with a total of 1665 PCOS patients and 1687 healthy subjects met the inclusion criteria and were included in this meta-analysis [7,14-26], with one study that included both *TNF-alpha (−308 G/A)* and *IL-6 (−174 G/C)* polymorphisms [22]. The flow chart in Figure 1 is a summary of the literature review process. Study characteristics are summarized in Table 1. Overall, six studies were conducted among Caucasians and eight among Asians. The Rotterdam diagnostic criteria were used in 12 studies and the NIH criteria in two. In 8 of 14 studies, DNA was extracted from peripheral blood and analyzed with a classic polymerase chain reaction–restriction fragment length polymorphism (PCR–RFLP) assay. The NOS results showed that the median overall score was 6 (range, 4–8). Thirteen studies were considered high quality and one as low quality. In addition, four studies reported a deviation of the genotype distributions among the controls from the HWE [17,21,23,25].

**Table 1:** **Characteristic of the studies included in this meta-analysis**

**Study name**	**Year**	**Country**	**Ethnicity**	**Age, years, mean ± SD**		**Sample size**		**PCOS diagnostic criteria**	**Genotyping method**	**NOS score**	**Genotype (case/control)**			***P***_**HWE**_^**b**^
				**Cases**	**Controls**	**Cases**	**Controls**				**11**	**12**	**22**	
*TNF-alpha (−308 G/A)*														
Milner et al. [14]	1999	Australia	Caucasian	NA	NA	84	108	NIH criteria	PCR-SSCP	4	59/63	23/42	2/3	0.194
Mao et al. [15]	2000	China	Asian	28.0 ± 0.5	31.1 ± 1.1	118	54	NIH criteria	PCR-RFLP	6	88/37	29/13	1/4	0.089
Vural et al. [22]	2010	Turkey	Caucasian	25 (17–39)^a^	27 (18–39)^a^	97	95	Rotterdam criteria	PCR-RFLP	8	78/77	16/15	3/3	0.055
Zhang et al. [16]	2010	China	Asian	29.0 ± 1.5	30.0 ± 1.5	78	40	Rotterdam criteria	Microarray	7	72/36	6/4	0/0	0.739
Deepika et al. [17]	2013	India	Asian	NA	NA	283	306	Rotterdam criteria	ARMS PCR	6	10/10	270/293	3/3	<0.05
Wen et al. [18]	2013	China	Asian	26.86 ± 4.5	27.3 ± 3.71	103	59	Rotterdam criteria	PCR-RFLP	8	89/52	14/7	0/0	0.628
Grech et al. [19]	2014	Greece	Caucasian	22.5 ± 3.2	NA	39	140	Rotterdam criteria	PCR-RFLP	5	33/125	6/14	0/1	0.394
*IL-6 (−174 G/C)*														
Walch et al. [20]	2004	Austria	Caucasian	28 (20–39)^a^	NA	62	94	Rotterdam criteria	Pyrosequencing	6	24/43	30/35	8/16	0.068
Erdogan et al. [21]	2009	Turkey	Caucasian	24.07 ± 1.32	25.01 ± 2.05	88	119	Rotterdam criteria	PCR-RFLP	8	57/32	26/75	5/12	<0.05
Vural et al. [22]	2010	Turkey	Caucasian	25 (17–39)^a^	27 (18–39)^a^	97	95	Rotterdam criteria	PCR-RFLP	8	59/46	34/42	4/7	0.536
Tumu et al. [23]	2013	India	Asian	26.35 ± 3.88	30.00 ± 5.17	104	156	Rotterdam criteria	Pyrosequencing	6	69/77	31/73	4/6	<0.05
*IL-1beta (−511 C/T)*														
Kolbus et al. [24]	2007	Austria	Caucasian	27.9 ± 5.0	28.8 ± 5.9	105	102	Rotterdam criteria	Pyrosequencing	7	43/40	47/48	15/14	0.947
Yang et al. [25]	2009	China	Asian	NA	NA	118	86	Rotterdam criteria	PCR-RFLP	5	30/34	56/26	32/26	<0.05
Mu et al. [26]	2010	China	Asian	26.91 ± 4.02	31.14 ± 4.22	200	177	Rotterdam criteria	PCR-RFLP	6	64/26	76/87	60/64	0.684
Xia et al. [7]	2013	China	Asian	29.75 ± 3.62	28.93 ± 3.91	59	56	Rotterdam criteria	PCR-RFLP	6	13/18	21/31	25/7	0.257

For *TNF-alpha (−308 G/A)*, 11 = GG, 12 = GA, 22 = AA; For *IL-6 (−174 G/C)*, 11 = GG, 12 = GC, 22 = CC; For *IL-1beta (−511 C/T)*, 11 = CC, 12 = CT, 22 = TT.

NA, not available; HWE, Hardy–Weinberg equilibrium; PCR-RFLP, polymerase chain reaction–restriction fragment length polymorphism; SSCP, single-strand conformational polymorphism; ARMS, amplification refractory mutation system; NOS, Newcastle-Ottawa Scale; ^a^values are given as median (range); ^b^ *p* value for HWE in controls.

### Meta-analysis results

**Figure 2:**
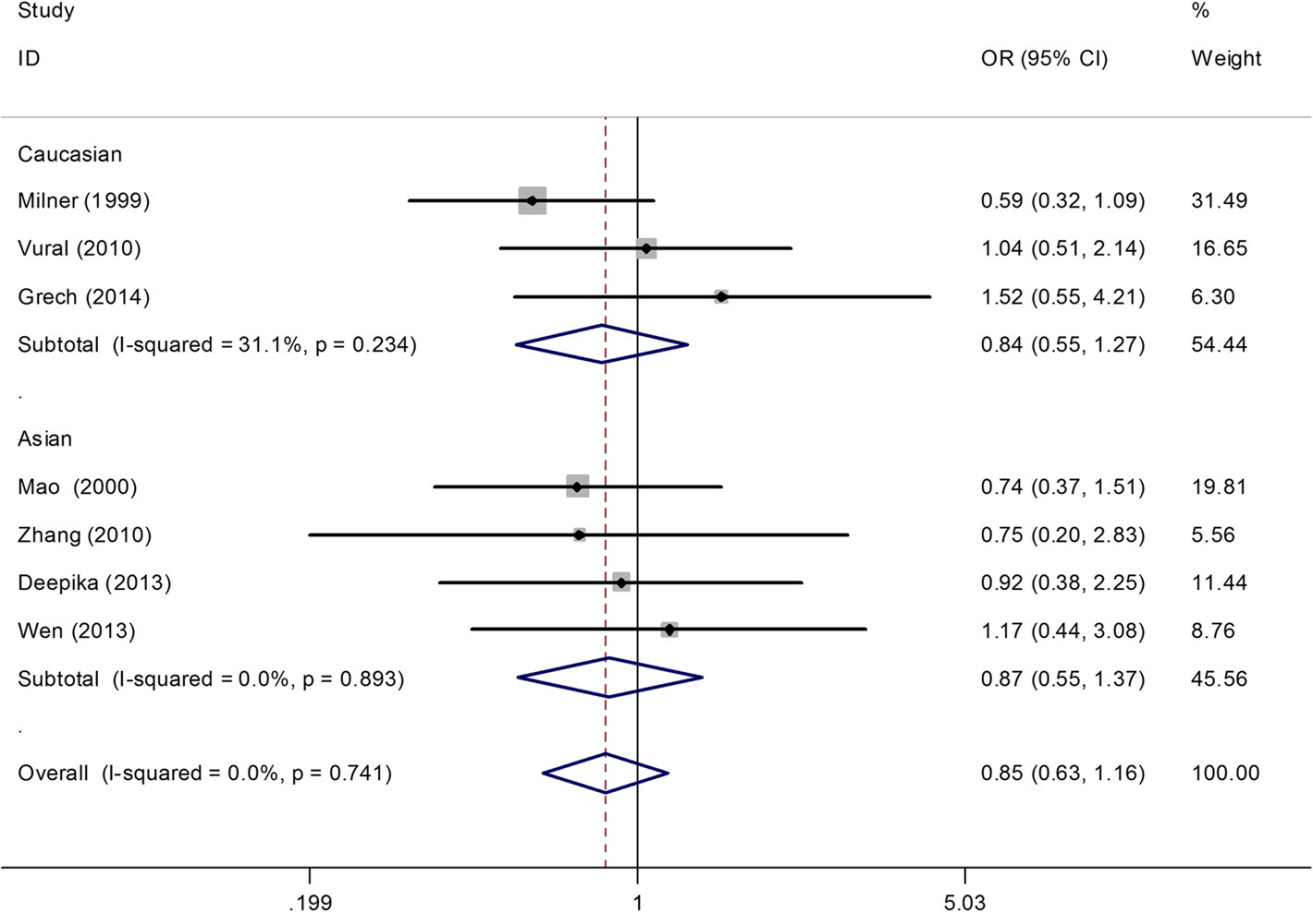
**Meta-analysis for the association between the** ***TNF-alpha (−308 G/A)*** **polymorphism and risk of polycystic ovary syndrome based on the dominant model (AA + GA vs. GG; stratified by ethnicity).**

**Figure 3:**
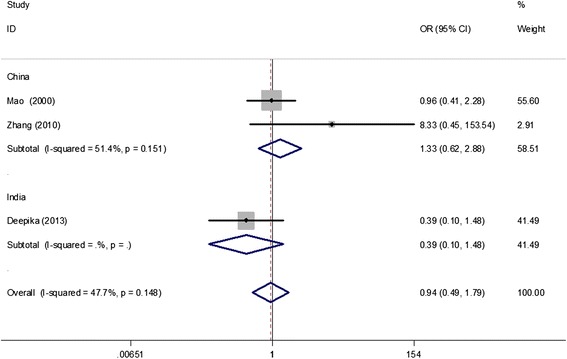
**A forest plot for the correlation of the** ***TNF-alpha (−308 G/A)*** **polymorphism with obesity in patients with polycystic ovary syndrome (BMI ≥ 25 kg/m** ^**2**^ **vs. BMI < 25 kg/m** ^**2**^ **) based on the dominant model (AA + GA vs. GG; stratified by country).**

A total of 802 cases and 802 controls were assessed to identify associations between the *TNF-alpha (−308 G/A)* polymorphism and PCOS. The A allele was considered as the risk variant. However, we found no association between the *TNF-alpha (−308 G/A)* polymorphism and PCOS risk in the overall population (A vs. G: OR = 0.93, 95% CI = 0.77–1.11; AA vs. GG: OR = 0.61, 95% CI = 0.26–1.41; GA vs. GG: OR = 0.89, 95% CI = 0.65–1.23; AA + GA vs. GG: OR = 0.85, 95% CI = 0.63–1.16; AA vs. GA + GG: OR = 0.66, 95% CI = 0.29–1.49). After performing stratified analysis for HWE in controls, sample size ≤ 200, PCR-RFLP, PCOS diagnosis criteria, and ethnicity separately, the association remained non-significant (Table 2, Figure 2). In addition, data on genotype distributions of the *TNF-alpha (−308 G/A)* polymorphism in PCOS patients with a body mass index (BMI) ≥ 25 kg/m^2^ and BMI < 25 kg/m^2^ were available in three studies [15-17]. The results showed no significant association of the *TNF-alpha (−308 G/A)* polymorphism with obesity in patients with PCOS (BMI ≥ 25 kg/m^2^ vs. BMI < 25 kg/m^2^) (AA + GA vs. GG: OR = 0.94, 95% CI = 0.49–1.79; Figure 3).

**Table 2:** **Meta-analysis of** ***TNF-alpha (−308 G/A)*** **polymorphism and PCOS risk**

**Comparison**	**No. of studies**	**A vs. G**		**AA vs. GG** ^**a**^		**GA vs. GG**		**AA + GA vs. GG**		**AA vs. GA + GG** ^**a**^	
		**OR (95% CI)**	***P***_**H**_	**OR (95% CI)**	***P***_**H**_	**OR (95% CI)**	***P***_**H**_	**OR (95% CI)**	***P***_**H**_	**OR (95% CI)**	***P***_**H**_
Overall	7	0.93 (0.77-1.11)^F^	0.610	0.61 (0.26-1.41)^F^	0.519	0.89 (0.65-1.23)^F^	0.725	0.85 (0.63-1.16)^F^	0.741	0.66 (0.29-1.49)^F^	0.495
HWE in controls/sample size ≤ 200	6	0.82 (0.61-1.10)^F^	0.634	0.53 (0.20-1.39)^F^	0.397	0.89 (0.63-1.25)^F^	0.603	0.84 (0.61-1.17)^F^	0.625	0.55 (0.21-1.44)^F^	0.389
PCR-RFLP	4	0.91 (0.63-1.32)^F^	0.469	0.47 (0.15-1.47)^F^	0.236	1.11 (0.72-1.71)^F^	0.864	1.00 (0.67-1.52)^F^	0.699	0.47 (0.15-1.46)^F^	0.245
Ethnicity											
Asian	4	0.95 (0.77-1.16)^F^	0.523	0.37 (0.10-1.36)^F^	0.124	0.96 (0.60-1.52)^F^	0.960	0.87 (0.55-1.37)^F^	0.893	0.39 (0.04-3.72)^R^	0.096
Caucasian	3	0.86 (0.59-1.24)^F^	0.354	0.89 (0.29-2.78)^F^	0.944	0.84 (0.54-1.30)^F^	0.201	0.84 (0.55-1.28)^F^	0.234	0.95 (0.31-2.95)^F^	0.984
PCOS diagnostic criteria											
NIH criteria	2	0.65 (0.44-1.02)^F^	0.869	0.31 (0.08-1.19)^F^	0.192	0.71 (0.44-1.13)^F^	0.345	0.65 (0.41-1.03)^F^	0.638	0.35 (0.09-1.30)^F^	0.154
Rotterdam criteria	5	1.01 (0.83-1.24)^F^	0.955	1.02 (0.33-3.19)^F^	0.991	1.08 (0.70-1.66)^F^	0.903	1.06 (0.70-1.62)^F^	0.930	1.05 (0.36-3.07)^F^	0.993

OR, odds ratio; CI, confidence interval; No., number; vs.: versus; *P*_H_, *p* value of Q-test for heterogeneity test; ^R^random-effect model; ^F^fixed-effect model.

^a^the studies by Zhang et al. and Wen et al. were not included since they presented 0 frequency of AA genotype in cases and controls.

**Figure 4:**
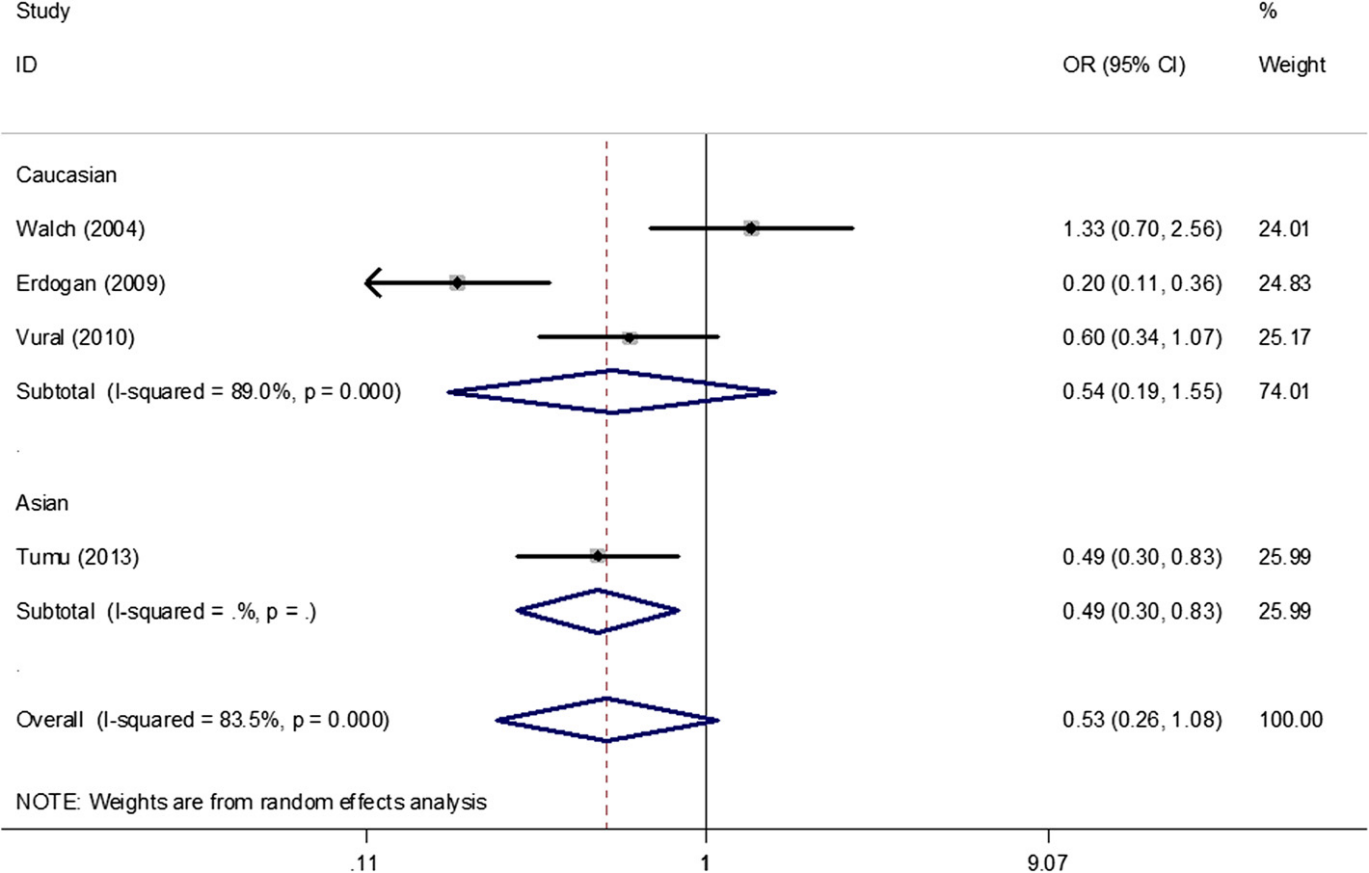
**Meta-analysis for the association between the** ***IL-6 (−174 G/C)*** **polymorphism and the risk of polycystic ovary syndrome based on the dominant model (CC + GC vs. GG; stratified by ethnicity).**

Four studies (351 cases and 464 controls) investigated the association of the *IL-6 (−174 G/C)* polymorphism with PCOS risk and were included in the analysis. Overall, no obvious associations were found by heterozygote comparison (GC vs. GG: OR = 0.54, 95% CI = 0.25–1.17) or in the dominant model (CC + GC vs. GG: OR = 0.53, 95% CI = 0.26–1.08) and recessive models (CC vs. GC + GG: OR = 0.67, 95% CI = 0.39–1.16) (Table 3, Figure 4). However, the results indicated that the *IL-6 (−174 G/C)* polymorphism was marginally associated with PCOS in the allelic model (C vs. G: OR = 0.63, 95% CI = 0.41–0.96) and by homozygote comparison (CC vs. GG: OR = 0.52, 95% CI = 0.30–0.93). In further subgroup analysis based on study sample size, the *IL-6 (−174 G/C)* polymorphism was significantly associated with a decreased PCOS risk among sample size > 200 in the allelic model (C vs. G: OR = 0.47, 95% CI = 0.28–0.80) and dominant model (CC + GC vs. GG: OR = 0.32, 95% CI = 0.13–0.78), as well as by homozygote comparison (CC vs. GG: OR = 0.38, 95% CI = 0.17–0.89) and heterozygote comparison (GC vs. GG: OR = 0.31, 95% CI = 0.13–0.74). Stratification by the genotyping method indicated that the examined SNP was associated with a decreased risk of PCOS among genotyping method of PCR-RFLP in the allelic model (C vs. G: OR = 0.49, 95% CI = 0.27–0.88) and by homozygote comparison (CC vs. GG: OR = 0.31, 95% CI = 0.13–0.72). Even so, the effect size was not significant among HWE in controls (sample size ≤ 200) and genotyping method of pyrosequencing under all genetic models.

**Table 3:** **Meta-analysis of** ***IL-6 (−174 G/C)*** **polymorphism and PCOS risk**

**Comparison**	**No. of studies**	**C vs. G**		**CC vs. GG**		**GC vs. GG**		**CC + GC vs. GG**		**CC vs. GC + GG**	
		**OR (95% CI)**	***P***_**H**_	**OR (95% CI)**	***P***_**H**_	**OR (95% CI)**	***P***_**H**_	**OR (95% CI)**	***P***_**H**_	**OR (95% CI)**	***P***_**H**_
Overall	4	0.63 (0.41-0.96)^R^	0.013	0.52 (0.30-0.93)^F^	0.329	0.54 (0.25-1.17)^R^	<0.01	0.53 (0.26-1.08)^R^	<0.01	0.67 (0.39-1.16)^F^	0.881
HWE in controls/sample size ≤ 200	2	0.84 (0.60-1.16)^F^	0.157	0.69 (0.32-1.50)^F^	0.398	0.97 (0.40-2.31)^R^	0.057	0.88 (0.41-1.92)^R^	0.074	0.65 (0.31-1.37)^F^	0.716
Sample size > 200^a^	2	0.47 (0.28-0.80)^R^	0.091	0.38 (0.17-0.89)^F^	0.189	0.31 (0.13-0.74)^R^	0.033	0.32 (0.13-0.78)^R^	0.074	0.69 (0.30-1.59)^F^	0.469
Caucasian	3	0.63 (0.34-1.17)^R^	<0.01	0.48 (0.26-0.91)^F^	0.211	0.57 (0.18-1.78)^R^	<0.01	0.54 (0.19-1.55)^R^	<0.01	0.61 (0.33-1.13)^F^	0.897
Genotyping method											
PCR-RFLP	2	0.49 (0.27-0.88)^R^	0.065	0.31 (0.13-0.72)^F^	0.461	0.35 (0.11-1.11)^R^	<0.01	0.35 (0.12-1.03)^R^	<0.01	0.54 (0.24-1.23)^F^	0.994
Pyrosequencing	2	0.80 (0.47-1.37)^R^	0.092	0.84 (0.38-1.84)^F^	0.824	0.83 (0.26-2.64)^R^	<0.01	0.80 (0.30-2.10)^R^	0.019	0.81 (0.38-1.70)^F^	0.687

OR, odds ratio; CI, confidence interval; No., number; vs.: versus; *P*_H_, *p* value of Q-test for heterogeneity test; ^R^random-effect model; ^F^fixed-effect model; ^a^deviated from HWE in controls.

**Figure 5:**
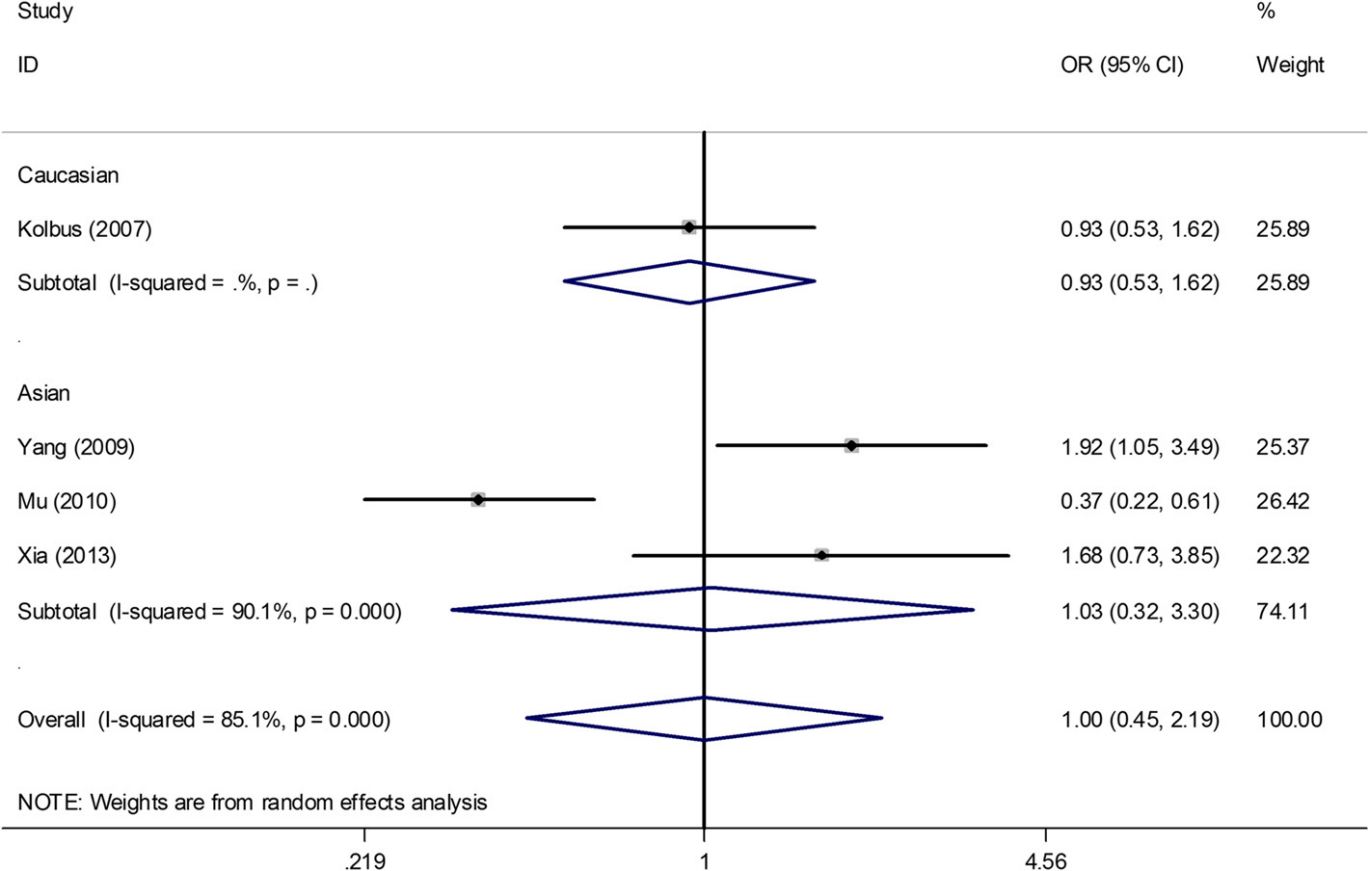
**Meta-analysis for the association between the** ***IL-1beta (−511 C/T)*** **polymorphism and the risk of polycystic ovary syndrome based on the dominant model (TT + CT vs. CC; stratified by ethnicity).**

**Figure 6:**
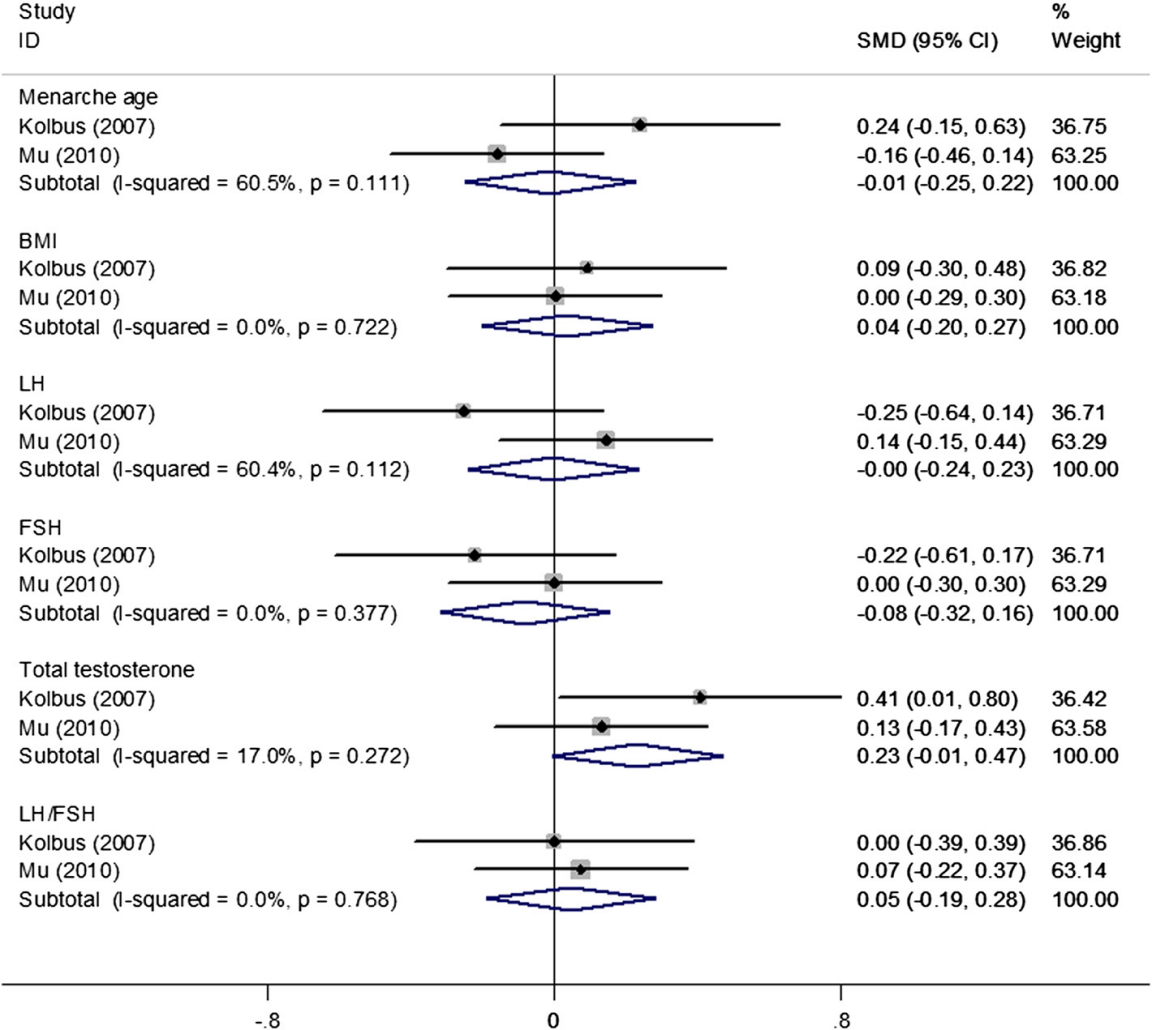
**A forest plot for the correlation of the** ***IL-1beta (−511 C/T)*** **polymorphism (TT + CT vs. CC) with several clinical and biochemical parameters in patients with polycystic ovary syndrome.**

Under all genetic models, no obvious associations were found between the *IL-1beta (−511 C/T)* polymorphism and PCOS risk when all studies were pooled into the meta-analysis (T vs. C: OR = 1.11, 95% CI = 0.67–1.84; TT vs. CC: OR = 0.89, 95% CI = 0.62–1.27; TC vs. CC: OR = 0.91, 95% CI = 0.40–2.11; TT + CT vs. CC: OR = 1.0, 95% CI = 0.45–2.19; TT vs. CT + CC: OR = 1.26, 95% CI = 0.62–2.53). In the subgroup analysis by ethnicity, HWE in controls, genotyping method, and study sample size, still no obvious associations were found (Table 4, Figure 5). Additionally, two studies [24,26] provided data between the *IL-1beta (−511 C/T)* polymorphism and several clinical and biochemical parameters in patients with PCOS. The results showed no significant association of the *IL-1beta (−511 C/T)* polymorphism (TT + CT vs. CC) with menarche age (SMD = −0.01, 95% CI = −0.25–0.22), BMI (SMD = 0.04, 95% CI = −0.20–0.27), and levels of luteinizing hormone (LH) (SMD = −0.00, 95% CI = −0.24–0.23), follicle-stimulating hormone (FSH) (SMD = −0.08, 95% CI = −0.32–0.16), total testosterone (SMD = 0.23, 95% CI = −0.01–0.47) and LH/FSH (SMD = 0.05, 95% CI = −0.19–0.28) in patients with PCOS (Figure 6).

**Table 4:** **Meta-analysis of** ***IL-1beta (−511 C/T)*** **polymorphism and PCOS risk**

**Comparison**	**No. of studies**	**T vs. C**		**TT vs. CC**		**TC vs. CC**		**TT + CT vs. CC**		**TT vs. CT + CC**	
		**OR (95% CI)**	***P***_**H**_	**OR (95% CI)**	***P***_**H**_	**OR (95% CI)**	***P***_**H**_	**OR (95% CI)**	***P***_**H**_	**OR (95% CI)**	***P***_**H**_
Overall	4	1.11 (0.67-1.84)^R^	<0.01	0.89 (0.62-1.27)^R^	<0.01	0.91 (0.40-2.11)^R^	<0.01	1.00 (0.45-2.19)^R^	<0.01	1.26 (0.62-2.53)^R^	<0.01
HWE in controls	3	1.08 (0.54-2.14)^R^	<0.01	1.16 (0.29-4.61)^R^	<0.01	0.65 (0.33-1.28)^R^	0.041	0.79 (0.34-1.86)^R^	<0.01	1.50 (0.53-4.25)^R^	<0.01
Asian/PCR-RFLP	3	1.17 (0.57-2.42)^R^	<0.01	1.29 (0.33-5.09)^R^	<0.01	0.92 (0.27-3.18)^R^	<0.01	1.03 (0.32-3.30)^R^	<0.01	1.37 (0.53-3.54)^R^	<0.01
Sample size > 200	3	0.90 (0.58-1.37)^R^	0.014	0.70 (0.47-1.03)^R^	0.014	0.91 (0.31-2.67)^R^	<0.01	0.86 (0.33-2.21)^R^	<0.01	0.83 (0.60-1.14)^F^	0.769

OR, odds ratio; CI, confidence interval; No., number; vs.: versus; *P*_H_, *p* value of Q-test for heterogeneity test; ^R^random-effect model; ^F^fixed-effect model.

### Heterogeneity test and sensitivity analysis

**Figure 7:**
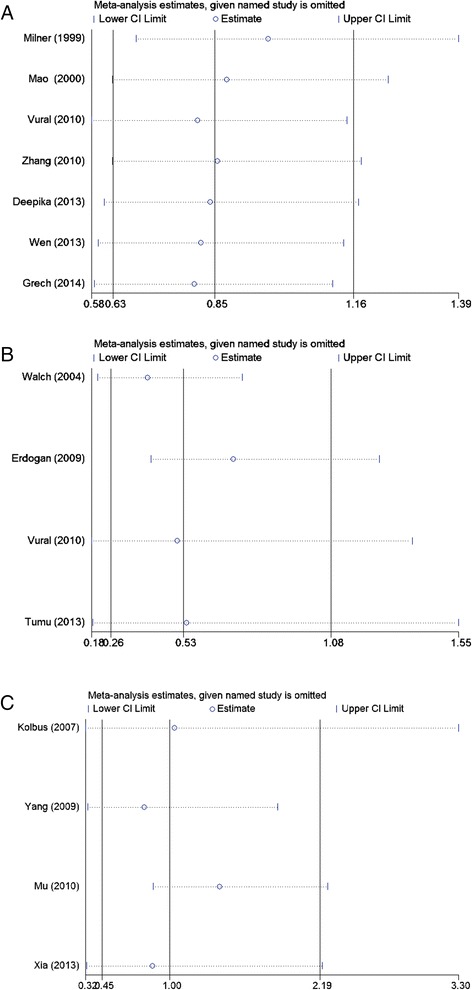
**Sensitivity analysis of the summary odds ratio (OR) coefficients on the associations among the** ***TNF-alpha (−308 G/A)*** **,** ***IL-6 (−174 G/C)*** **and** ***IL-1beta (−511 C/T)*** **polymorphisms with the risk of polycystic ovary syndrome based on the dominant model. (A)**, *TNF-alpha (−308 G/A)*; **(B)**, *IL-6 (−174 G/C)*; **(C)**, *IL-1beta (−511 C/T)*.

Regarding the *TNF-alpha (−308 G/A)* polymorphism and PCOS, there was no heterogeneity among studies in overall comparisons (*p* > 0.10). For the *IL-6 (−174 G/C)* and *IL-1beta (−511 C/T)* polymorphisms and PCOS, there was statistical significance with between-study heterogeneity (*p* < 0.10). To explore sources of heterogeneity across studies, subgroup analyses by ethnicity, HWE in controls, genotyping method, and study sample size were conducted. However, none of these variables could explain the heterogeneity. To evaluate the stability of the results of the meta-analysis, sensitivity analyses of the omitted individual studies were performed sequentially. This procedure confirmed that our results were reliable and robust (Figure 7).

### Publication bias

Publication bias was assessed by both the Begg’s test and Egger’s test. The statistical results did not show any evidence of publication bias (*p* > 0.05), with the exception of the *IL-1beta (−511 C/T)* polymorphism under the allelic model with the Egger's test (*p* = 0.023, Table 5). Sensitivity analysis using the trim and fill method was then performed to assess the possibility of publication bias. However, no trimming was performed and the data were unchanged throughout the filled meta-analysis, which suggested the absence of publication bias for the *IL-1beta (−511 C/T)* polymorphism under the allelic model.

**Table 5:** **Statistical analyses of publication bias for** ***TNF-alpha*** **,** ***IL-6*** **and** ***IL-1beta*** **gene polymorphisms**

**Category**	**Allele model**	**Homozygote comparison**	**Heterozygote comparison**	**Dominant model**	**Recessive model**
*TNF-alpha (−308 G/A)*					
Begg’s test	1.00	0.462	0.368	0.133	0.221
Egger’s test	0.704	0.732	0.190	0.180	0.616
*IL-6 (−174 G/C)*					
Begg’s test	0.308	1.00	0.734	0.734	0.734
Egger’s test	0.496	0.729	0.583	0.665	0.906
*IL-1beta (−511 C/T)*					
Begg’s test	0.308	0.308	0.734	0.308	0.089
Egger’s test	0.023	0.089	0.532	0.346	0.178

## Discussion

It is well known that PCOS is a proinflammatory state and chronic low-grade inflammation was found to promote the development of metabolic disruption and ovarian dysfunction in PCOS [34]. Moreover, variants in genes encoding several proinflammatory cytokines and their receptors associated with obesity, insulin resistance, and diabetes have also been found to be associated with PCOS [17,35]. As common multifunctional cytokines, TNF-alpha, IL-6, and IL-1 have been proposed to influence the processes of ovulation, fertilization, and implantation, which are also affected in women with PCOS [36]. However, no significant difference was found by meta-analysis of serum TNF-alpha and IL-6 concentrations in women with PCOS and controls, although only nine studies of TNF-alpha and 10 studies of IL-6 were included [37]. The occurrence of polymorphisms in the *TNF-alpha*, *IL-6*, and *IL-1* genes in women affected by PCOS has also been investigated over the past few decades [22,24]. However, due to the relative small sample size, no clear consensus has been reached. The aim of the present study was to perform a meta-analysis of the best evidence available in an attempt to provide high-quality data on the linkage between *TNF-alpha (−308 G/A)*, *IL-6 (−174 G/C)*, and *IL-1beta (−511 C/T)* polymorphisms and PCOS risk.

To the best of our knowledge, this is the first meta-analysis concerning *TNF-alpha (−308 G/A)*, *IL-6 (−174 G/C)*, and *IL-1beta (−511 C/T)* polymorphisms and PCOS risk. Based on our results, no significant association was found between *TNF-alpha (−308 G/A)* and *IL-1beta (−511 C/T)* polymorphisms and PCOS risk in the overall population or subgroup analysis under all genetic models. For the *IL-6 (−174 G/C)* polymorphism, however, variant C allele was associated with a lower risk of PCOS in the allelic model (C vs. G: OR = 0.63, 95% CI = 0.41–0.96) and by homozygote comparison (CC vs. GG: OR = 0.52, 95% CI = 0.30–0.93), even though the *p-*value (0.032 and 0.042, respectively) was marginal. Our results indicate that the *IL-6 (−174 G/C)* polymorphism likely conveys a protective effect against PCOS. A possible explanation for this finding is that the *IL-6* C allele results in low IL-6 production, which may normalize ovarian function. However, the results of this meta-analysis should be cautiously interpreted. First, in the subgroup analysis, no association was found between the *IL-6 (−174 G/C)* polymorphism and PCOS risk by the genotyping method of pyrosequencing in any genetic model. Although the *IL-6 (−174 G/C)* polymorphism was associated with PCOS in Caucasian populations and in the subgroup analysis of PCR-RFLP genotyping method, one study [21] showed deviations in genotype frequency from the HWE of controls. Second, after excluding two studies with deviations from the HWE in controls [21,23], which had a sample size of > 200, the result showed that the *IL-6 (−174 G/C)* polymorphism was no longer associated with PCOS. Although the relatively limited study number (only two studies for *IL-6 (−174 G/C)* polymorphism in the subgroup analysis specific to HWE in controls and genotyping method of pyrosequencing) and small sample size might have resulted in the null results, we could not rule out the possibility that the *IL-6 (−174 G/C)* polymorphism was actually not associated with PCOS risk. Our overall positive results might be dominated by the studies showing deviation from the HWE in controls. No heterogeneity was observed for the *TNF-alpha (−308 G/A)* polymorphism. For the *IL-6 (−174 G/C)* and *IL-1beta (−511 C/T)* polymorphisms, however, there was significant heterogeneity in the overall population and in the subgroup of HWE in controls, ethnicity, genotyping method, and sample size that might have influenced the results. Sensitivity analysis was then conducted, which confirmed the stability and reliability of our results.

The pooled results also demonstrated that there was no obvious association of the *TNF-alpha (−308 G/A)* polymorphism with obesity, or between the *IL-1beta (−511 C/T)* polymorphism with several clinical and biochemical parameters, in patients with PCOS. However, It should be noted that not all studies evaluated the effect of interactions, with two studies [17,26] providing complete data on different genetic models and three [15,16,24] just presenting data on the dominant model. Furthermore, other clinical and laboratory characteristics (including metabolic parameters and androgen parameters) according to the genotypes of *TNF-alpha (−308 G/A)*, *IL-6 (−174 G/C)*, and *IL-1beta (−511 C/T)* polymorphisms in the PCOS patients and the control group could not evaluated due to a lack of sufficient data. Therefore, our results, which only presented data on the dominant model of the *TNF-alpha (−308 G/A)* and *IL-1beta (−511 C/T)* polymorphisms, should be interpreted with caution. Insulin resistance and hyperinsulinemia are the central features of the metabolic disturbances typical for PCOS [37]. The *TNF-alpha (−308 G/A)* polymorphism was reported to be associated with altered responses to oral glucose tolerance testing in the PCOS group [14]. TNF-alpha is over-expressed in the adipose tissue of obese subjects in proportion to the degree of insulin resistance [38]. The *IL-6* polymorphism investigated in this study was shown to be associated with lipid abnormalities and impaired insulin sensitivity [39,40]. IL-1beta was proposed as a promoter of nitrogen (NO) generation and apoptosis of pancreatic islet B cell, which also induced insulin resistance [26]. However, a study by Puder et al. showed that an increase in both low-grade chronic inflammation and insulin resistance in PCOS patients is associated with increased central fat excess rather than PCOS status [41]. It remains to be established whether the proinflammatory state in PCOS is primarily a result of genetic variation or simply inflamed adipose tissue, because there is an increased prevalence of abdominal adiposity in PCOS across all weight classes. In fact, no differences were reported in levels of TNF-alpha, IL-6, and markers of inflammation between obese women with PCOS and obese controls [42]. A study by Mohlig et al. also showed that plasma levels of IL-6 and C-reactive protein were not increased in women with PCOS when compared to controls matched by age and BMI [43]. Previous studies have proposed that polymorphisms to the *TNF-alpha (−308 G/A)* and *IL-1beta (−511 C/T)* genes might be the genetic basis for the increase in TNF-alpha and IL-1beta serum levels in patients with PCOS [7,24]. Regarding the *IL-6 (−174 G/C)* polymorphism, however, several investigations have reported that the homozygous CC genotype was associated with higher serum IL-6 levels, which is in contrast to studies indicating that the G allele is associated with increased IL-6 secretion in subjects with PCOS, metabolic syndrome, and insulin resistance [12,22,39,44]. It appears that transcriptional control of IL-6 is multifaceted and may be dependent on the impact of other sites of polymorphisms which may be in linkage disequilibrium with the *IL-6* gene locus [45]. Furthermore, it has been suggested that obesity-associated genes, environmental factors, and gene–environment interactions, as well as dietary habits are also responsible for the increasing prevalence of PCOS worldwide [17,46,47].

Several limitations to this study need to be taken into account when interpreting our results. First, the number of study and the sample sizes were relatively small for analysis of each gene polymorphism thereby having insufficient power to detect any true difference between cases and controls. Second, heterogeneity was detected in the *IL-1beta (−511 C/T)* polymorphisms and the source of heterogeneity could not be identified due to the limited number of studies included in each pooled outcome. Third, the results of our meta-analysis were not adjusted for confounding factors, such as age and BMI, due to the lack of sufficient data, which might have influenced the effect estimates. Finally, the etiology of PCOS has long been described as multifactorial; however, it was difficult for us to analyze interactions between genes, lifestyles, and certain environmental factors.

## Conclusions

In summary, the results of this meta-analysis suggested that variations of the *TNF-alpha (−308 G/A)*, *IL-6 (−174 G/C)*, and *IL-1beta (−511 C/T)* genes might not represent genetic risk factors for PCOS. However, there is a need for further larger-scale studies, including other loci of the *TNF-alpha*, *IL-6*, and *IL-1beta* genes, to confirm our results and elucidate the potential association and influence of cytokine gene polymorphisms, and gene–environment interactions on the development of PCOS.

## Additional file

Additional file 1:
**Prisma checklist.**

## Abbreviations

BMI: Body mass index
CI: Confidence interval
HWE: Hardy–Weinberg equilibrium
IL: Interleukin
OR: Odds ratio
*p*: Probability
PCOS: Polycystic ovary syndrome
PRISMA: The preferred reporting items for systematic reviews and meta-analysis
SNP: Single nucleotide polymorphism
TNF-alpha: Tumor necrosis factor alpha
vs.: Versus
